# Supplementary material for: Interplay of valley, layer and band topology towards interacting quantum phases in moiré bilayer graphene
Source: Nat Commun. 2024 Jul 28;15:6351. doi: 10.1038/s41467-024-50475-x (PMC11284233; doi:10.1038/s41467-024-50475-x)
Supplement: Supplementary file 1 — Supplementary information [file 41467_2024_50475_MOESM1_ESM.pdf]

Supplementary Information:

Interplay of valley, layer and band topology towards interacting quantum phases in moiré bilayer graphene

Yungi Jeong, Hangyeol Park, Taeho Kim, K. Watanabe, T. Taniguchi, Jeil Jung, and Joonho Jang

Correspondence to: joonho.jang@snu.ac.kr

Supplementary Note 1. Additional measurement data

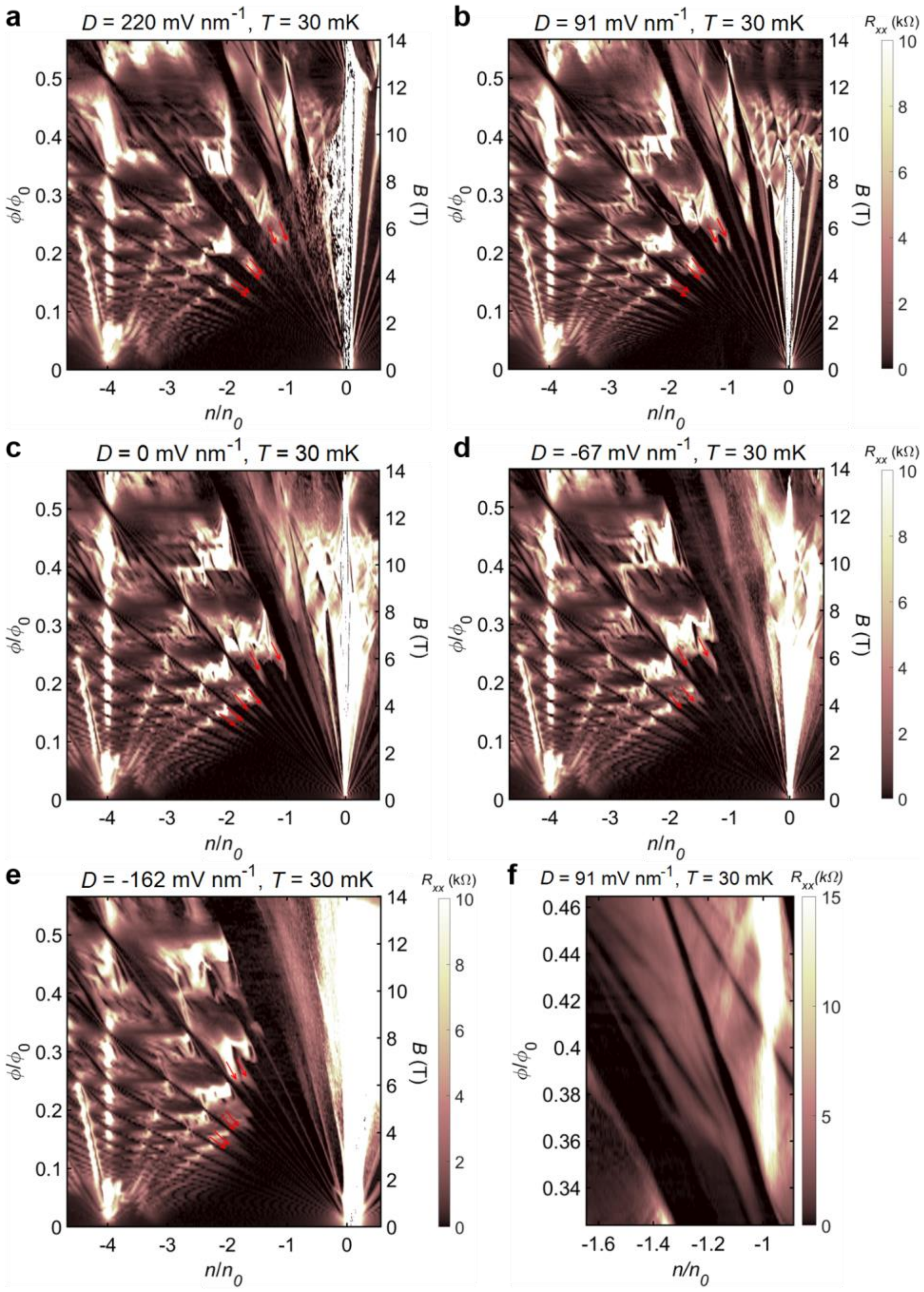

Supplementary Figure 1 | Landau fan diagram with various D fields. Longitudinal resistance versus carrier density and magnetic field at **a,** D = 220 $mV\ nm^{-1}$, **b,** D = 91 $mV\ nm^{-1}$ (higher resolution Fig.), **c,** D = 0, **d,** D = -67 $mV\ nm^{-1}$, and **e,** D = -162 $mV\ nm^{-1}$. Red arrows indicate metallic states that strongly feel the moiré potential within each $N \geq 2$ Landau level. As the D field is reduced from **b** to **e**, it can be seen that the positions indicated by the arrows move as shown in Fig. 3 of the main text. **f,** A more closely zoomed in image of Fig. 5a. Each color scale is truncated at the end value of its respective colorbar.

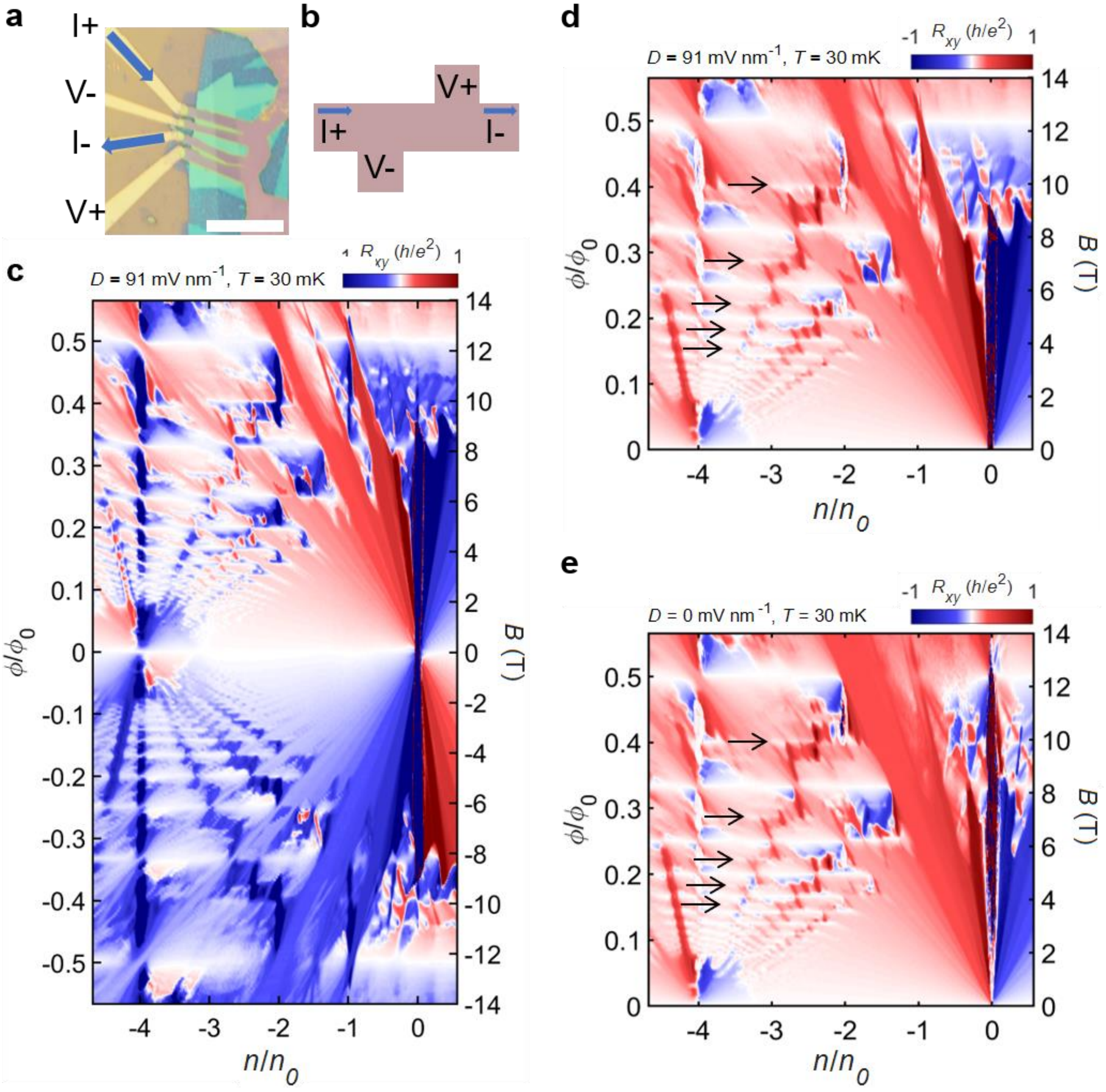

Supplementary Figure 2 | Hall measurement a, Hall measurement configuration. Current flows from I+ electrode to I- electrode and voltage (V+)-(V-) was measured. Scale bar, 15 μm. **b,** Schematic of the measurement configuration. Unfolding the horseshoe-shaped sample, it can be seen that **b** is topologically equivalent to **a**. And from this, we can expect a negative residual R_{xx} component in the measurement. Especially, this effect is strong for vertical features at n/n_0 = -1,-2,and -4 because these are not expected to have a chiral edge state. **c,** Hall resistance as a function of normalized carrier density and magnetic flux at D = 91 $mV\ nm^{-1}$. **d–e,** Plot of $(R_{xy}(n,B) - R_{xy}(n,-B))/2$. at **d,** D = 91 $mV\ nm^{-1}$ and **e,** D=0. The residual R_{xx} component was removed by subtracting the measured value at the opposite magnetic field. This makes blue vertical lines have values close to zero as expected (t = 0). And each incompressible state has a quantized Hall resistance depending on the value of t, with a corresponding slope in the fan diagram. Along the

$\phi/\phi_0 = 1/q$ lines, the first order Brown–Zak oscillation occurs and the sign of R_{xy} is reversed as the effective magnetic field felt by the BZ quasiparticle is reversed. In addition, horizontal lines with R_{xy} close to zero are observed at the higher order BZ oscillation ($\phi/\phi_0 = 2/q$) lines (Black arrows in **d**,**e**).

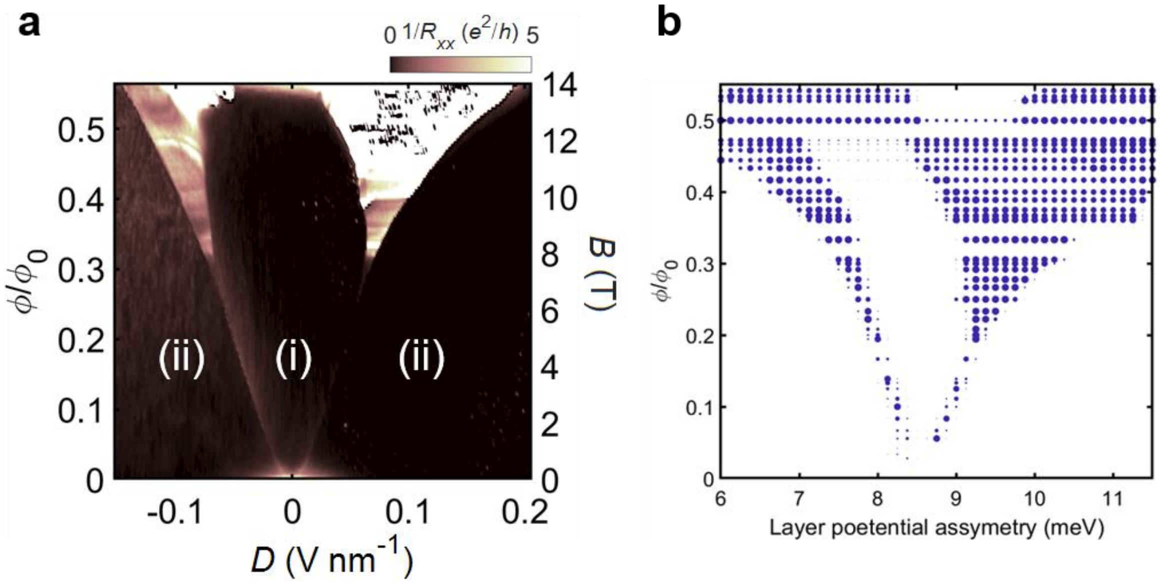

Supplementary Figure 3 | Displacement field dependent CNP states a, Reciprocal of Longitudinal conductance in units of e^2/h as a function of applied perpendicular electric displacement field and magnetic field at n = 0 (CNP) and T = 30 mK. Labels (i) and (ii) indicate the two different insulating $\nu = 0$ phases; (i) canted antiferromagnet state and (ii) layer polarized insulator state known in pristine BBG.[1] There is a phase transition point between the two phases where the conductance increases. But, as the magnetic field increases, this phase transition point widens and a metallic region appears between (i) and (ii). **b,** A numerical simulation of gap closings (with the marker size inversely proportional to the gap size) near CNP for the same conditions is presented for comparison, showing a good agreement with the measurement. This gap closing is likely due to the overlapping of the broadened conduction and valence bands under the influence of the moiré potential.

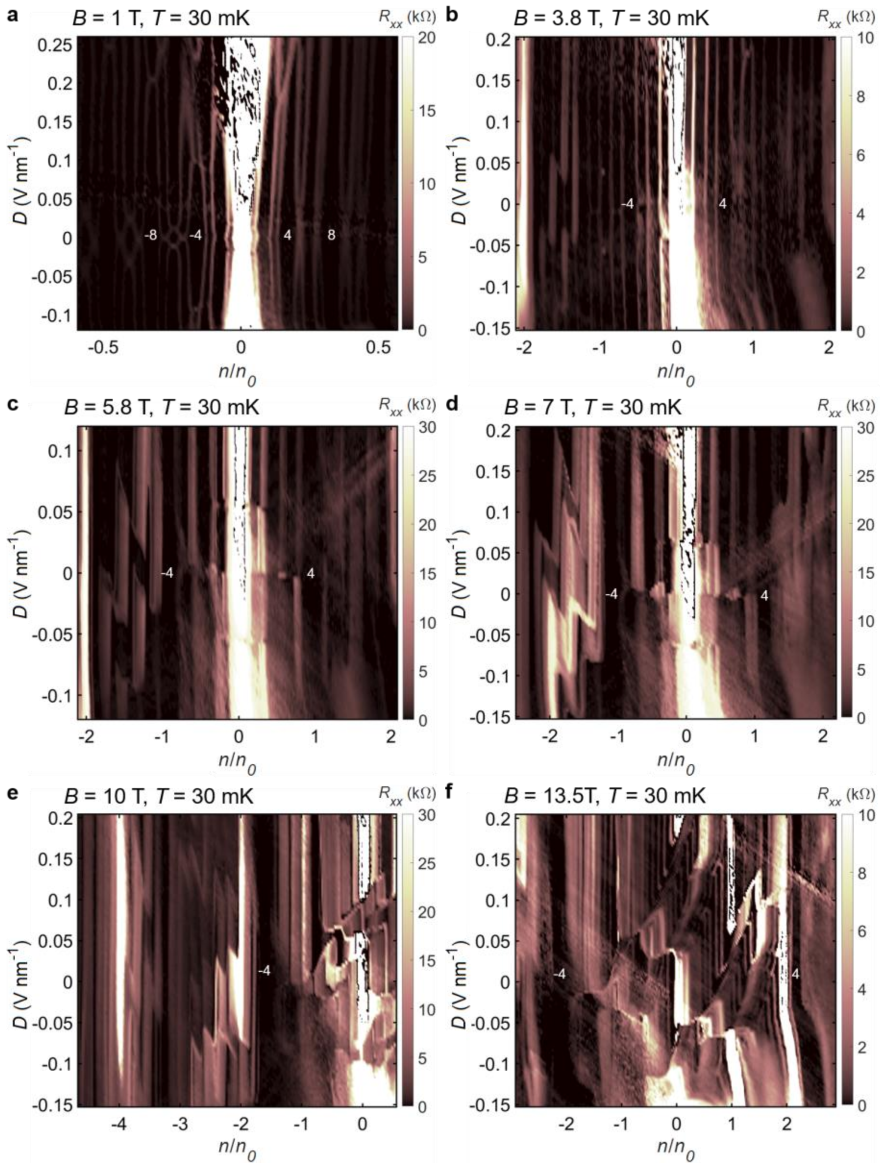

Supplementary Figure 4 | $n - D$ sweep at various magnetic field. Longitudinal resistance versus carrier density and D field at **a,** B = 1 T, **b,** B = 3.8 T, **c,** B = 5.8 T, **d,** B = 7 T, **e,** B = 10 T, and **f,** B = 13.5 T. Numbers in each figure represent the Landau filling factor ν. **a** shows that at the low magnetic field, there is little effect from the moiré potential and a single particle Landau level spectrum of intrinsic BBGs with broken spin–valley degeneracy appears. Around D = 0, intra-Landau level crossings for each N were observed, and for large D field, inter-Landau level crossings between different Ns also occurred.[2] **b** is the zoomed-out image of Fig. **3a**. Two of four N = 3 LL feels the moiré potential while $N \leq 2$ LLs are not yet affected by the moiré potential. In **c**,**d**, it is observed that the states with strongly feeling moiré potentials in the ZLL and N = 2 LL shift in opposite directions as D is varied. **e**,**f** shows that a large number of CIs undergo complex transitions, and the insulating gap at CNP closes in a certain D field range. Each color scale is truncated at the end value of its respective colorbar.

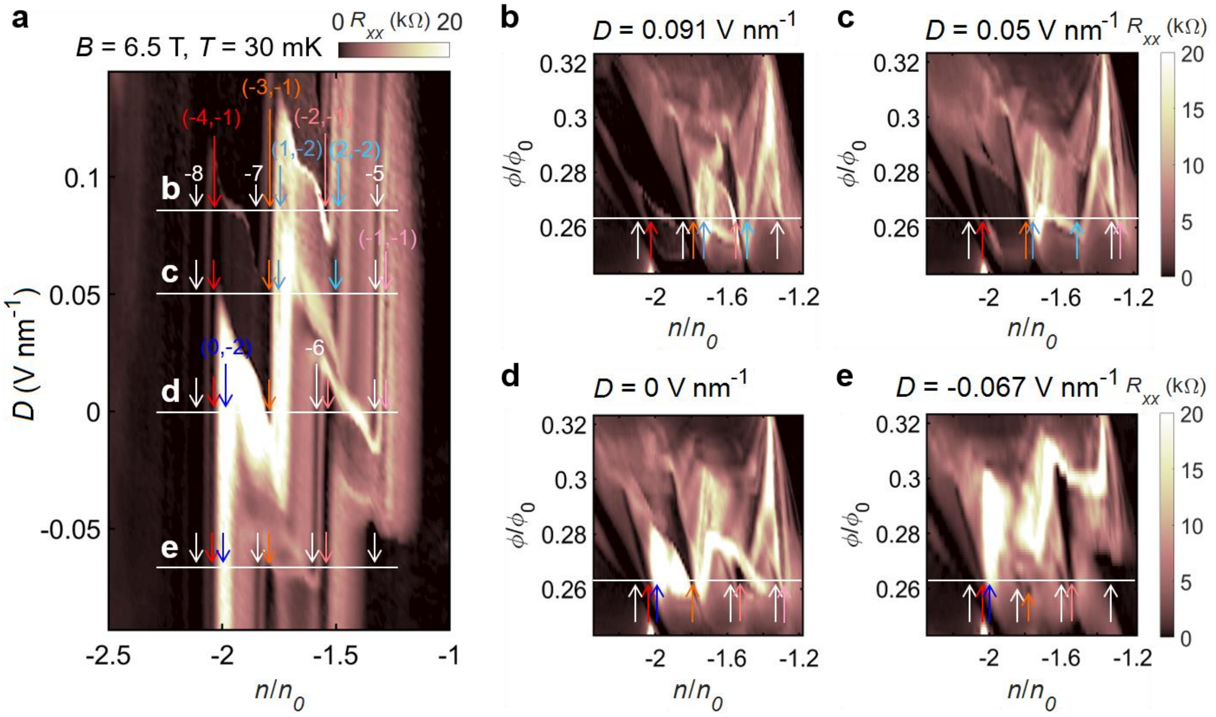

Supplementary Figure 5 | Displacement field tuning of Chern insulator states. a, Longitudinal resistance versus carrier density and displacement field at B = 6.5 T ($\phi/\phi_0 = 0.263$), T = 30 mK which is the same region as in **Fig.3f**. Each white horizontal line corresponds to a white horizontal line in **b**–**e** and each incompressible state(t,s) is pointed by the same colored arrows as in **Fig.3g** (s is omitted for IQHE states). **b–e,** Local Landau fan diagram of longitudinal resistance at different perpendicular displacement fields. Each state is represented by a line segment satisfying the Diophantine equation in the Landau fan diagram by the corresponding (t,s). Each color scale is truncated at the end value of its respective colorbar.

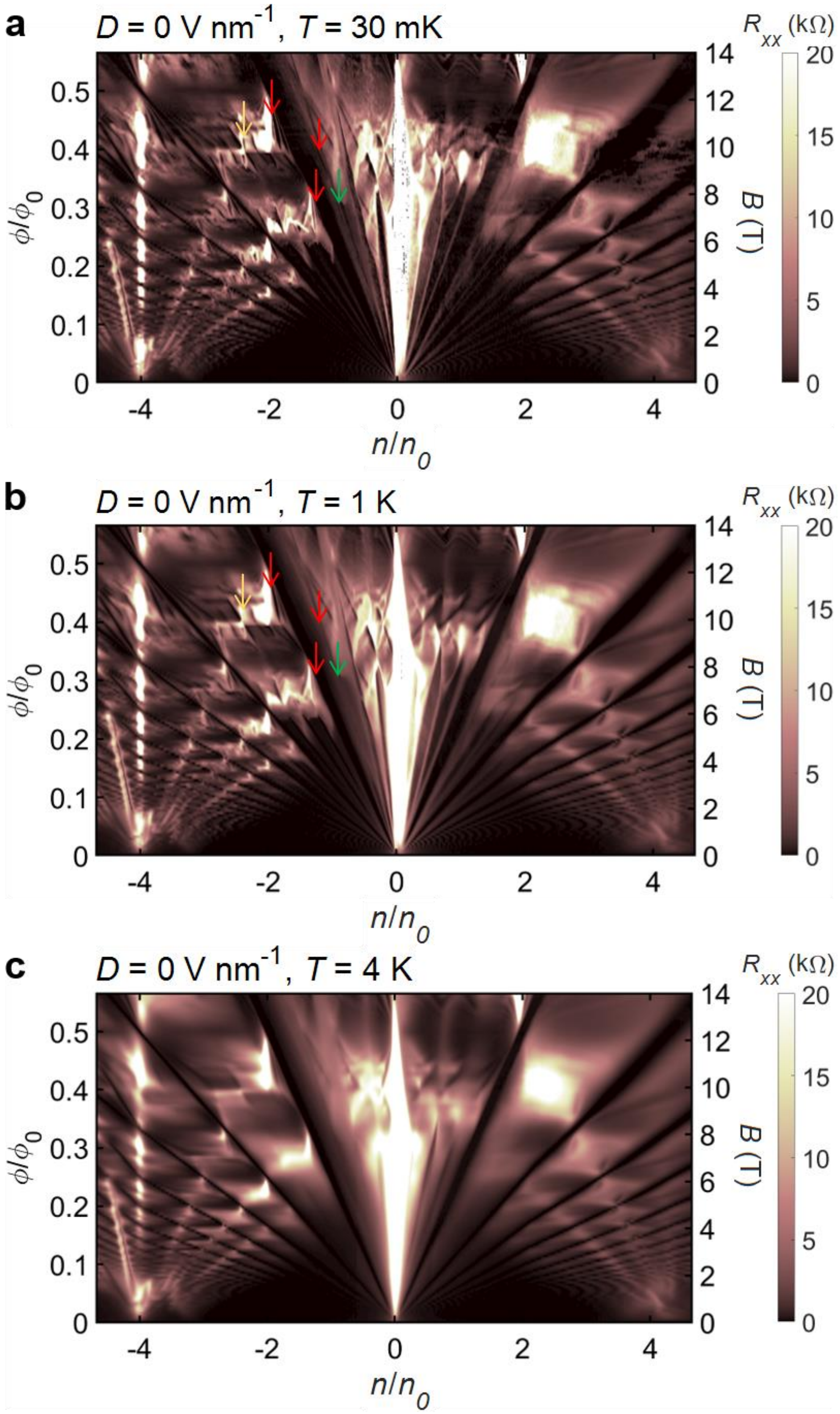

Supplementary Figure 6 | Landau fan diagram with various temperatures at *D* = 0. Longitudinal resistance versus carrier density and magnetic field at **a,** *T* = 30 mK, **b,** *T* = 1 K, and **c,** *T* = 4 K. Each color scale is truncated at the end value of its respective colorbar. At *T* = 1 K, the CI (yellow arrow), SBCI (red arrow), and FQHE (green arrow) states disappeared. And at *T* = 4 K, most of the CIs have melted away.

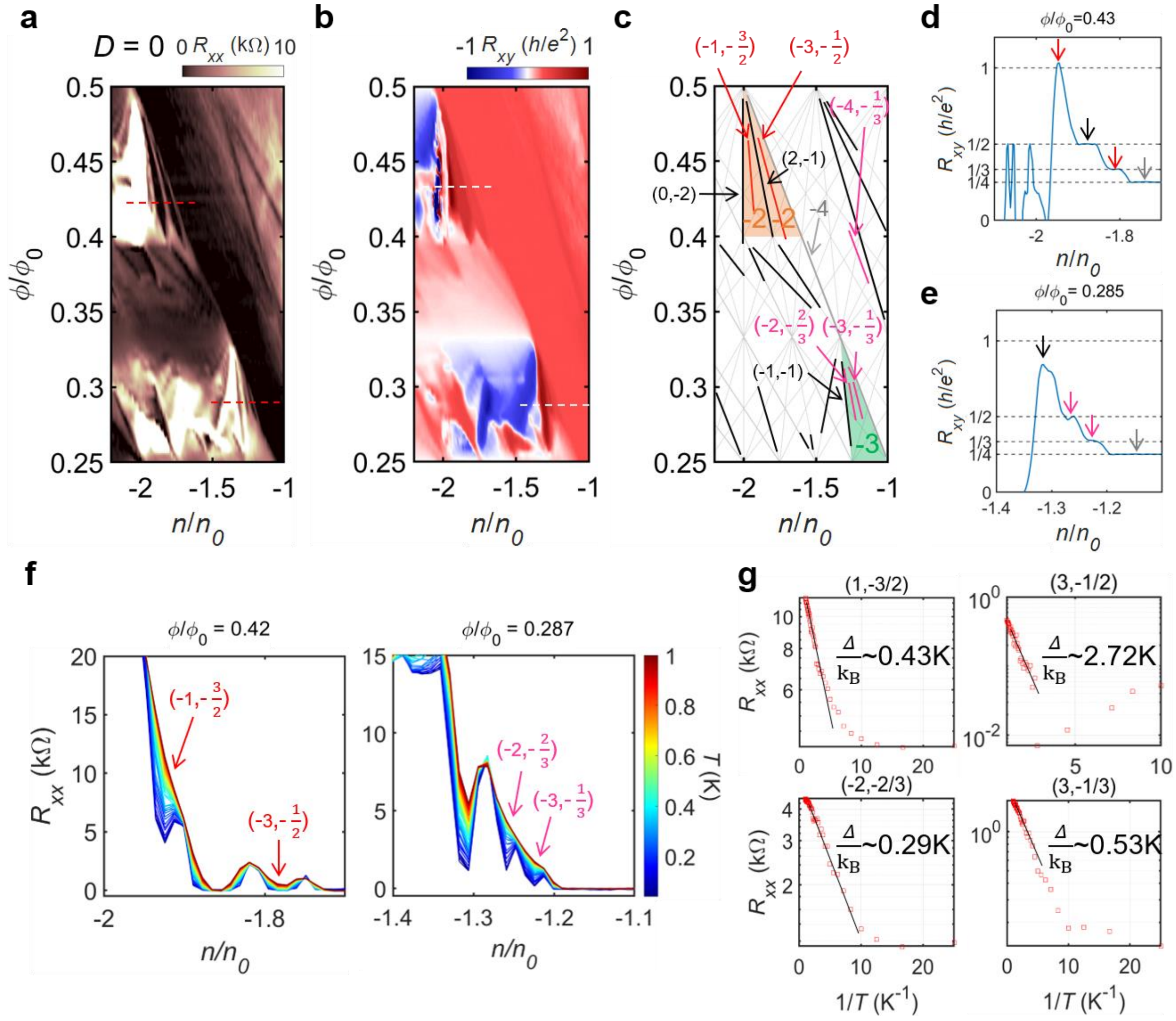

Supplementary Figure 7 | Symmetry broken Chern insulator states in higher Landau level at zero displacement field. a,b A zoomed in image of longitudinal(Hall) resistance Landau fan diagram at $D = 0$ (See **Supplementary Figure 1c** and **2e**). The color scale is truncated at 10 kΩ in **a**. **c,** Wannier diagram of the same area as **a, b**. The upper SBCI has (t,s) = (-1,-3/2), (-3,-1/2), from left to right, and the lower SBCI has (-2,-2/3), (-3,-1/3), respectively. The upper SBCIs are half-filled with the band with $\boldsymbol{\Delta} t$ = -2, and the lower SBCIs are 1/3 and 2/3 filled with the band with $\boldsymbol{\Delta} t$ = -3, respectively. **d–e,** Line cuts along the upper (**d**), lower (**e**) dashed line in **b**. The colored arrows indicate the states corresponding to the same colored lines in **c**. Each of the incompressible states showed a well quantized Hall resistance except the CI (-1, -1) state in **e** which didn't reached h/e^2. This is probably due to a part of the R_{xx} component (bright metallic part surrounding the gap) was not removed during the removal process, or the measurement speed didn't allow the value to saturate as the t value flipped from 2 to -1. **f,** Temperature dependence of longitudinal resistance along the dashed lines in **a**. **g,** Arrhenius plots of identified SBCIs.

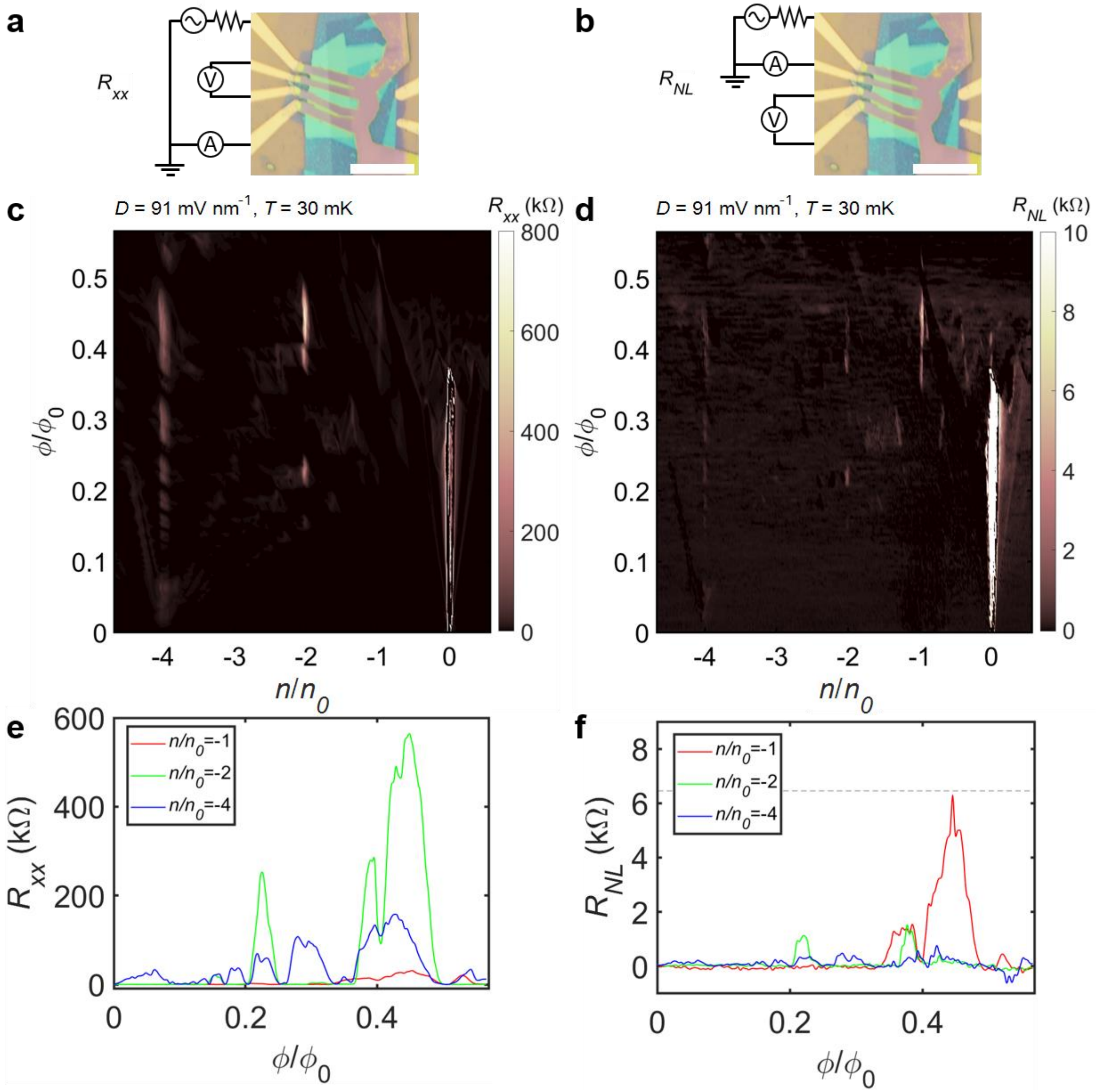

Supplementary Figure 8 | Nonlocal measurement of helical edge states at t=0 insulators. a–b, Measurement configuration of longitudinal (R_{xx}, **a**) and non-local (R_{NL}, **b**) resistance. Scale bar, 15 μm. **c–d,** Longitudinal (**c**) and non-local (**d**) resistance, as a function of normalized carrier density and magnetic flux, measured at T = 30 mK with $D = 91 mV\ nm^{-1}$. **e–f,** Line cuts of longitudinal (**e**) and non-local (**f**) resistance along the magnetic field direction at n/n_0 = -1 (red), -2 (green), and -4 (blue). Dashed horizontal line in **f** represents $h/4e^2$.

Supplementary Note 2. Device fabrication

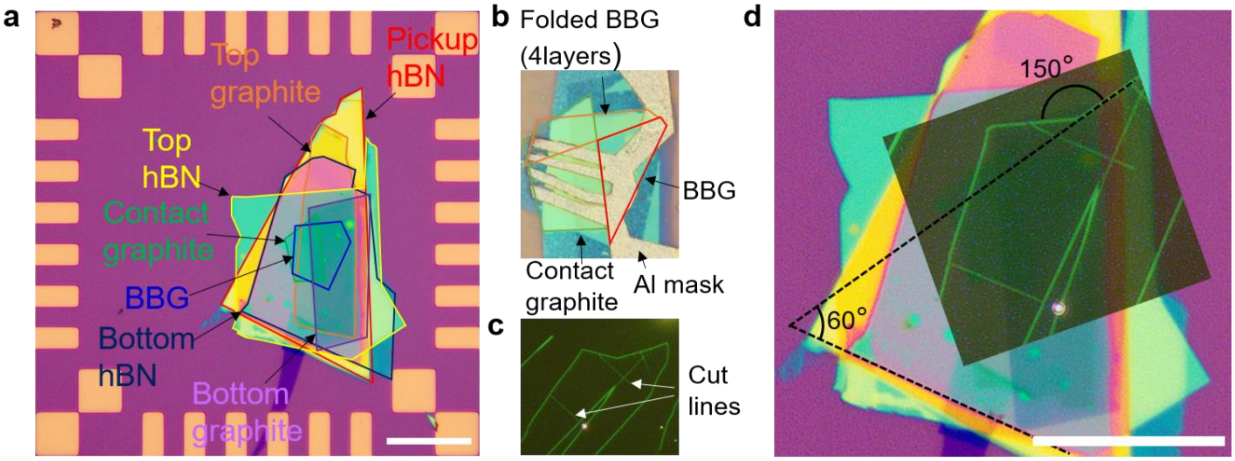

Supplementary Figure 9 | BBG/hBN aligned heterostructure a, Optical microscope image of the heterostructure stack. Each component is outlined in a different color and indicated by an arrow. **b,** Zoomed-in image of the stack during the reactive ion etching(RIE) process. Since graphene and hBN have a very high selectivity for CF_4 etching, the contrast between the graphene-protected and non-graphene-protected regions during RIE is dramatic, making it easy to see the alignment of the BBG and hBN. **c,** Dark field image of the BBG flake used in fabrication. A long BBG flake was cut by the scanning probe based local anodic oxidation method.[3] **d,** Overlapped image of **a** and **c**. **b** was used for alignment. It can be seen that one crystal axis of BBG is aligned at 60 degrees to the crystal axis of the bottom hBN. Scale bar, 30 μm.

Supplementary Note 3. Transport data axis normalization and superlattice size estimation.

The x-axis of the raw data in the Landau fan diagram is the gate voltage proportional to the carrier density, and the y-axis is the perpendicular magnetic field. To convert this to superlattice filling factor and normalized flux density, top gate and bottom gate sweep and Hall measurement were performed at low field. The carrier density n and vertical electric displacement field D can be expressed as follows using the top gate voltage V_t and bottom gate voltage V_b.

$$n = \frac{c_t V_t + c_b V_b}{e} = \frac{c}{e}\{(\cos\theta)V_t + (\sin\theta)V_b\} \equiv \frac{c}{e}V_x$$

$$D = \frac{-c_t V_t + c_b V_b}{2\epsilon_0} = \frac{c}{2\epsilon_0}\{-(\cos\theta)V_t + (\sin\theta)V_b\} \equiv \frac{c}{2\epsilon_0}V_y$$

$$c = \sqrt{{c_t}^2 + {c_b}^2}, \qquad \theta = \mathrm{atan}\left(\frac{c_b}{c_t}\right)$$

Where e is the unit charge, ϵ_0 is the permittivity of vacuum, c_t and c_b are the capacitance per unit area between the sample and the top gate and bottom gate respectively. If we think of (c_t, c_b) as a single two-dimensional vector and call the norm of this vector c and the polar angle θ, we can define V_x, the voltage proportional to the carrier density, and V_y, the voltage proportional to the D field, as above. Then, if we know θ and c, we can find the carrier density and D field from the voltage. First, to measure θ, a longitudinal resistance measurement was performed by sweeping the top gate voltage and bottom gate voltage in the magnetic field of 0.5T.

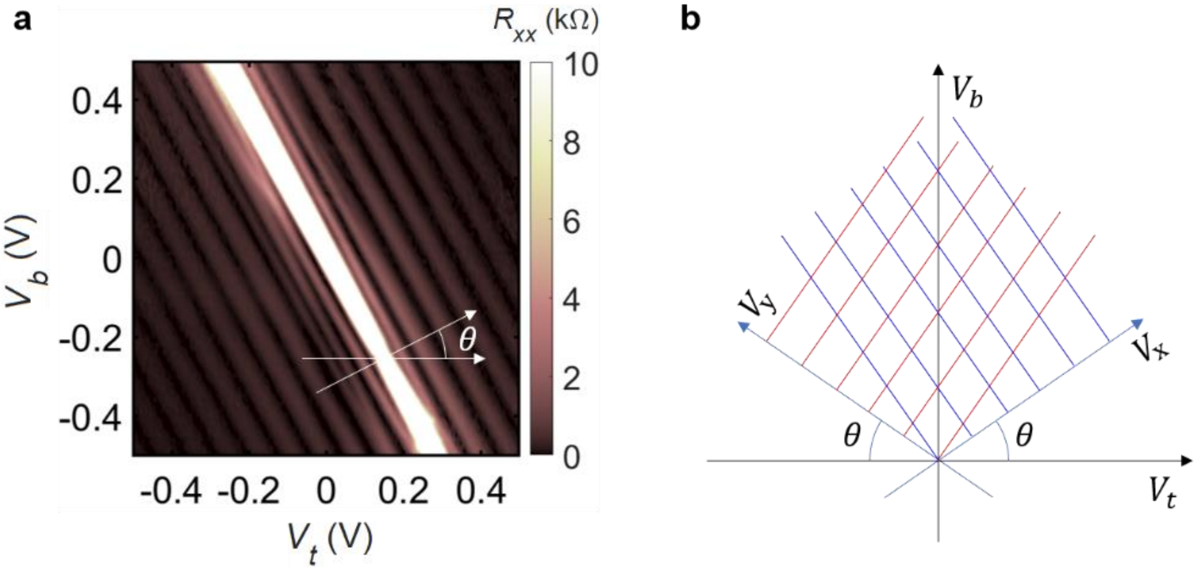

Supplementary Figure 10 | Capacitance ratio determination. a, Longitudinal resistance was measured by sweeping the top and bottom gate voltage at $B = 0.5$ T, $T = 30$ mK. Bright white diagonal line at the center is the CNP of BBG and each black line represents an integer quantum Hall state. By measuring the angle between V_t direction and $\mathrm{grad}(n)$ direction, we can get the ratio between the top and bottom capacitance. θ was 0.505 rad in our device. **b,** Schematic diagram of axis conversion. Blue and red lines perpendicular to V_x and V_y are the equipotential line of each variable. θ is the angle defined the same as in **a**.

The darker colored regions with lower resistance are the integer quantum Hall states with chiral edge modes, and the lighter regions in between are CNP (brightest line) and the metallic states with partially filled Landau level extended states. The direction perpendicular to these diagonal lines is the direction of increasing carrier density, and by measuring the angle between this direction and the x-axis (V_t axis), θ defined above can be obtained, and θ = 0.505rad was measured. From this, it can be seen that $c_b = 0.5528c_t$. By fixing V_y, D field can be fixed and only the carrier density can be tuned. And to measure c, low field Hall measurement was performed

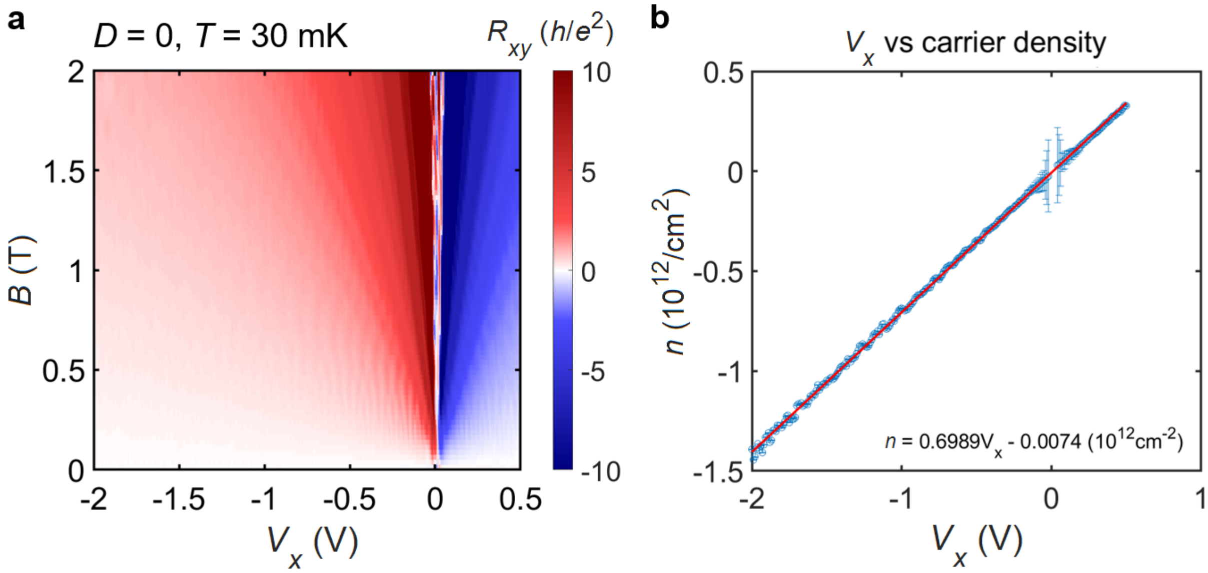

Supplementary Figure 11 | Capacitance size determination. a, Low field Hall resistance measurement. In the low carrier density region, quantum Hall is already visible below 1 T, but as the density increases, R_{xy} shows a linear relationship as predicted by the Drude model. **b,** Measured V_x dependence of the carrier density. From the Drude model, $R_{xy}(B) = -\frac{B}{ne}$. So the slope of $R_{xy}(B)$ is proportional to the reciprocal of the carrier density, which is calculated and plotted for each V_x. Error bars are proportional to the slope error of $R_{xy}(B)$ at each V_x. From the linear fit, we can see that $n = 0.6989V_x - 0.0074\ (10^{12}\ cm^{-2})$, and using the fact that the coefficient of V_x is c/e, we can see that $c\ =\ 1.1198 \times 10^{-15}\ F\ \mu m^{-2}$.

The Hall measurement was performed in the configuration shown in **Supplementary Figure 2a**, and since it is not an ideal Hall bar geometry, it measures a mixed value of R_{xx} and R_{xy}, but at the Drude model level, R_{xx} is independent of the magnetic field, so the slope can be used to determine the carrier density. From the measurement results, we determined the relationship between carrier density n and V_x.

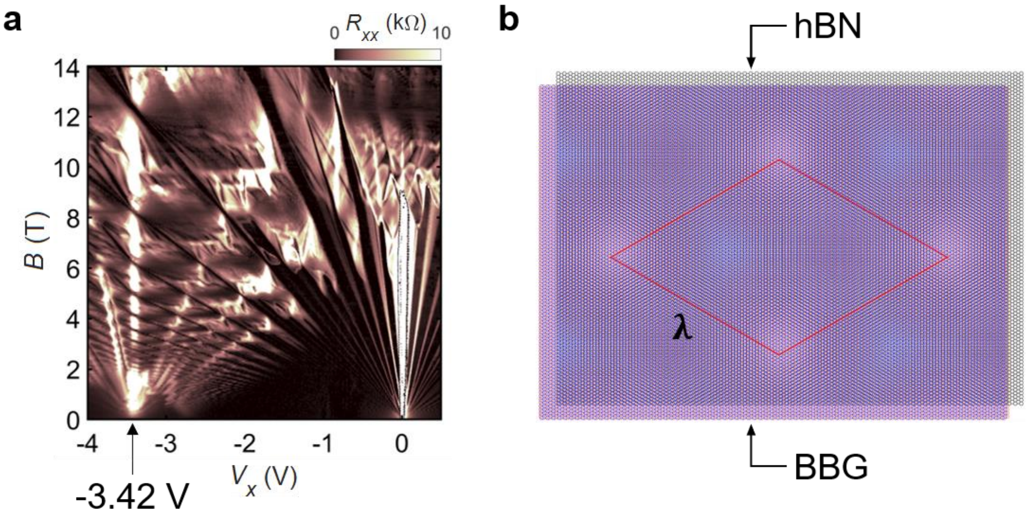

Supplementary Figure 12 | Determination of superlattice lattice parameter. a, Raw data of the Landau fan diagram. Longitudinal resistance was measured as V_x and B were swept. Superlattice band insulator was observed at $V_x = -3.42\ V$. This corresponds to the carrier density $n = 2.397 \times 10^{12}\ cm^{-2}$. **b,** moiré superlattice pattern of 0 degree aligned BBG/hBN heterostructure. The lattice constant mismatch of about 1.8% generates a triangular superlattice structure with the lattice constant λ.

From **Supplementary Figure 12**, we can see that a band insulator appears at $V_x = -3.42\ V$. This can occur when the moiré superlattice is filled with all four flavors of spin and valley. The superlattice parameter λ can be calculated from the corresponding carrier density and the fact that the moiré superlattice is a triangular lattice.

$$n_0 = \left(\frac{\sqrt{3}}{2}\lambda^2\right)^{-1}$$

$$4n_0 = 2.397 \times 10^{12}\ \mathrm{cm}^{-2} \ \Rightarrow\ \lambda = 13.88\ \mathrm{nm}$$

From the magnitude of the superlattice parameter, we can see that our sample has BBG and hBN aligned at almost 0 deg.

Alternatively, the superlattice parameter can be obtained independently by observing the Brown–Zak oscillation. From **Fig. 2** and **Supplementary Figure 2**, $\phi/\phi_0 = 1/q$ line is equivalent to $B = (24.7/q)\ T$ line, so we can estimate the superlattice unit cell area by $A \times 24.7T = \phi_0 = h/e$, $A = \frac{\sqrt{3}}{2}\lambda^2$. The resulting λ is 13.90 nm which is almost the same value obtained by the above method.

Supplementary Note 4. Numerical simulation of Hofstadter energy spectra and Wannier plots of the BBG/hBN superlattice with zero-degree alignment

After electronic energy eigenvalues are obtained by numerically diagonalizing at various perpendicular magnetic fields, we transform it to the Wannier plot as a function of density and magnetic field as in **Supplementary Figure 13**. It shows features that can be directly associated with our measured data. First of all, in the low magnetic fields, the LL band broadening due to the moiré potential is selectively strong for a certain valley (see also **Supplementary Figure 15**). As discussed in the main text, it is counterintuitive that the valley degrees of freedom determine the strength of the superlattice effect, not the layer degrees of freedom.

The multiple plots by varying the interlayer potential difference in **Supplementary Figure 14** — where we define D field = (interlayer potential difference) / (interlayer distance) — shows the pronounced features that strongly depend on the D field. We reproduce the insulating gaps at the full fillings, the characteristic spectra of the ZLL and the valley degrees of freedom's dependence on the external vertical electric displacement field, etc. Importantly, the vertical insulating phases at $n/n_0 = -2$ and -4 measured in **Fig. 4** of the main text are either absent or having energy gaps just similar to other incompressible Chern phases in the calculation. This thus supports our conclusion that the strong insulating phase at $n/n_0 = 2$ has an enhanced energy gap due to the electron correlation, and it is likely the consequence of the narrow bandwidth of the isolated valence band (see the calculated spectra in **Supplementary Figure 13a–d**), as discussed in the main text.

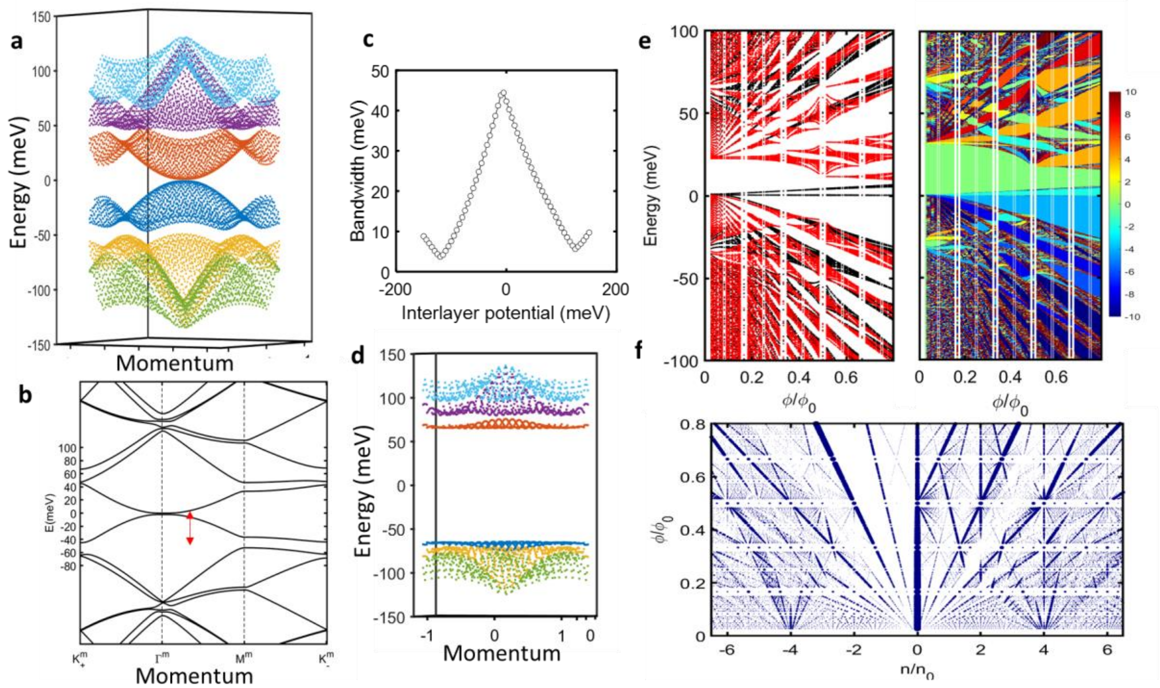

Supplementary Figure 13 | Hofstadter spectra and Wannier plots as a function of the interlayer potential difference. a–b, Zero magnetic field spectra as a function of momentum in the moiré Brillouin zone. **c,** The bandwidth calculated for the first valence band as a function of the interlayer potential. Interestingly, the band even becomes nearly flat in a strong D field, while not accessible in our current sample. **d**, The energy spectra near the minimum bandwidth in **c**. **e**, Magnetic field induced Hofstadter spectra at interlayer potential $U_{layer} = 0$. Two distinct valleys are indicated in black (K) and red (K') colors, and the Chern numbers for energy gaps are indicated with colors. **f**, Wannier diagram calculated from **e**. The size of the gaps are represented with the size of a marker, and only energy gaps larger than 0.1 meV are plotted. Note that spin degrees of freedom are assumed to be degenerated.

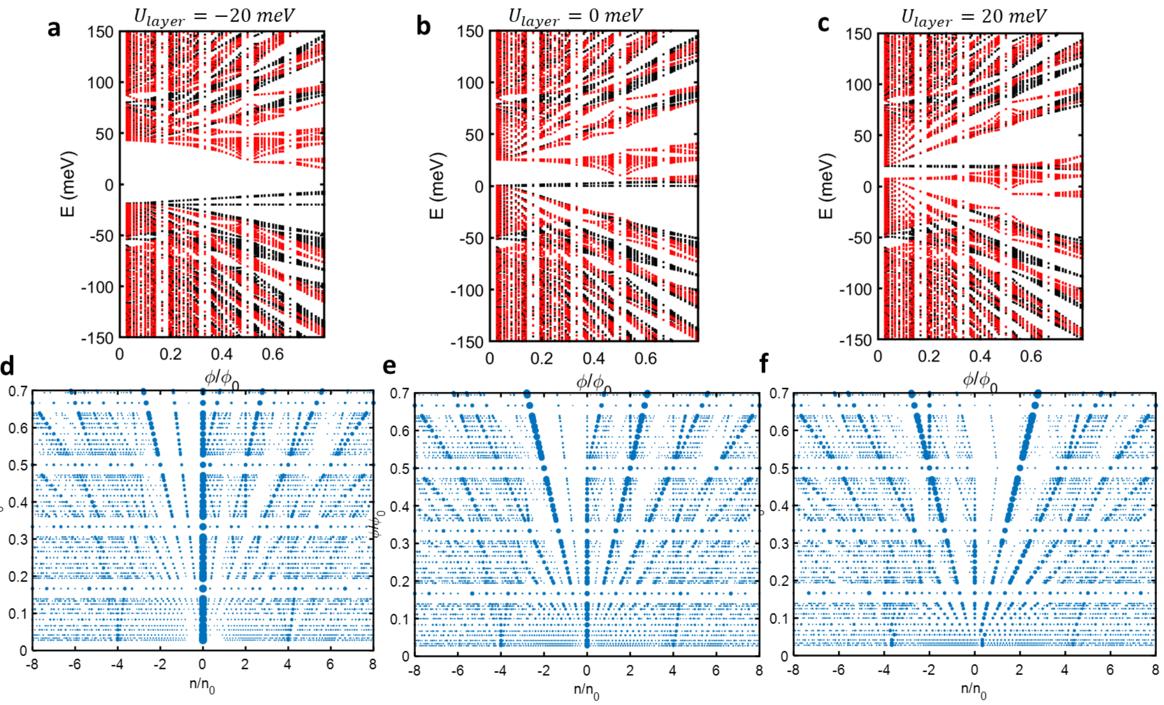

Supplementary Figure 14 | Electron energy spectra and Wannier plots for three different interlayer potential differences. a–c, In the spectra, the valleys are plotted in black (K) and red (K') colors separately. **d–f**, The Wannier plots only show energy gaps whose values are larger than 0.1 meV.

Supplementary Note 5. Numerical simulation of the correlation of valley, layer and Landau level index of the BBG/hBN superlattice

Based on the continuum model simulation of the previous section, we further calculated the valley-dependent energy spectra as a function of the interlayer potential at a fixed magnetic field. First of all, due to the dominant cyclotron energy scale, the LLs are sharply defined in the case of intrinsic BBG, and the moiré-broadened LLs are still well-resolved for the BBG with aligned hBN. The valley-dependent bands are plotted separately in blue (K) and red (K') colors. From the intra-LL energy crossings of valley-dependent bands (**Supplementary Figure 15b**), we clearly see a peculiar correlation between the valleys, layers and LL indices to support the conclusion drawn based on **Equation (1)** in the main text. We further show the filling sequence of bands based on the energy spectra and, in **Supplementary Figure 15d**, obtain qualitatively similar to our transport measurement to **Fig. 3**. Most strikingly to us, the simulation results clearly confirm that the effects of the moiré superlattice from the hBN interface are selectively felt by one of the valleys (namely K'), and we attribute this behavior to be the cause for the striking "fork" features (i.e. LL subbands with high longitudinal resistance in the Landau fan) discussed in the main text. In the following section, we further discuss the possible topological origin of the valley-selective effects based on the simulation.

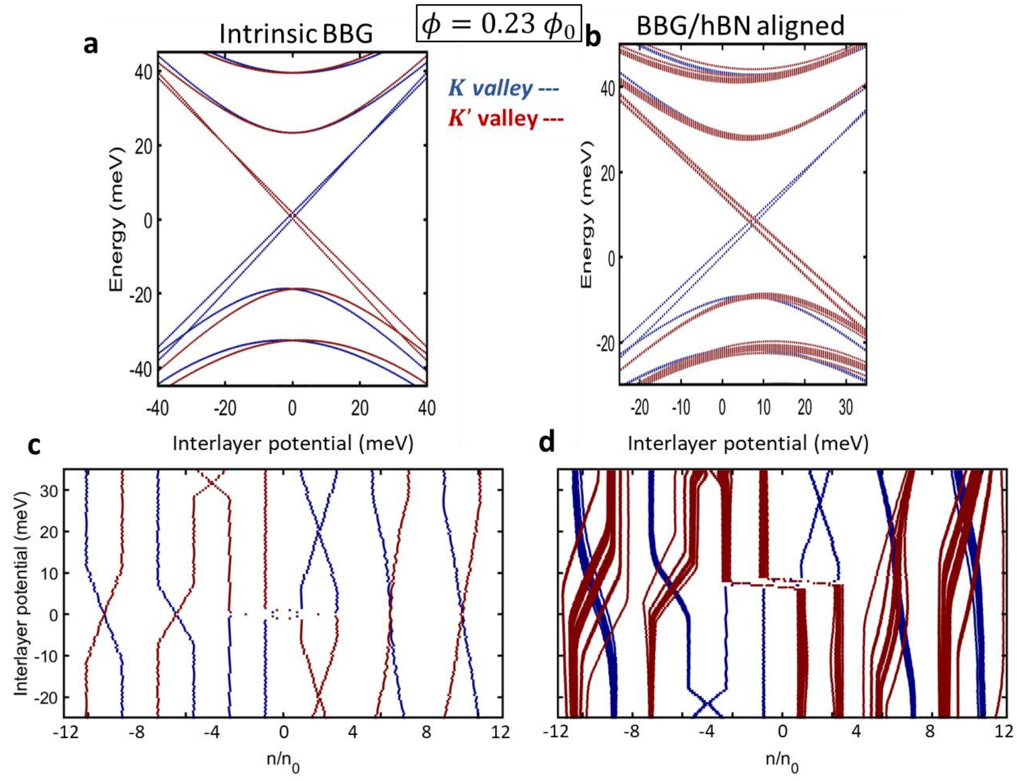

Supplementary Figure 15 | Electronic energy spectra and filling sequences calculated based on a continuum model, as explained in the text, for an intrinsic BBG (**a** and **c**) and an BBG aligned with hBN (**b** and **d**), as a function of the interlayer potential difference and density at $\phi = 0.23\phi_0$.

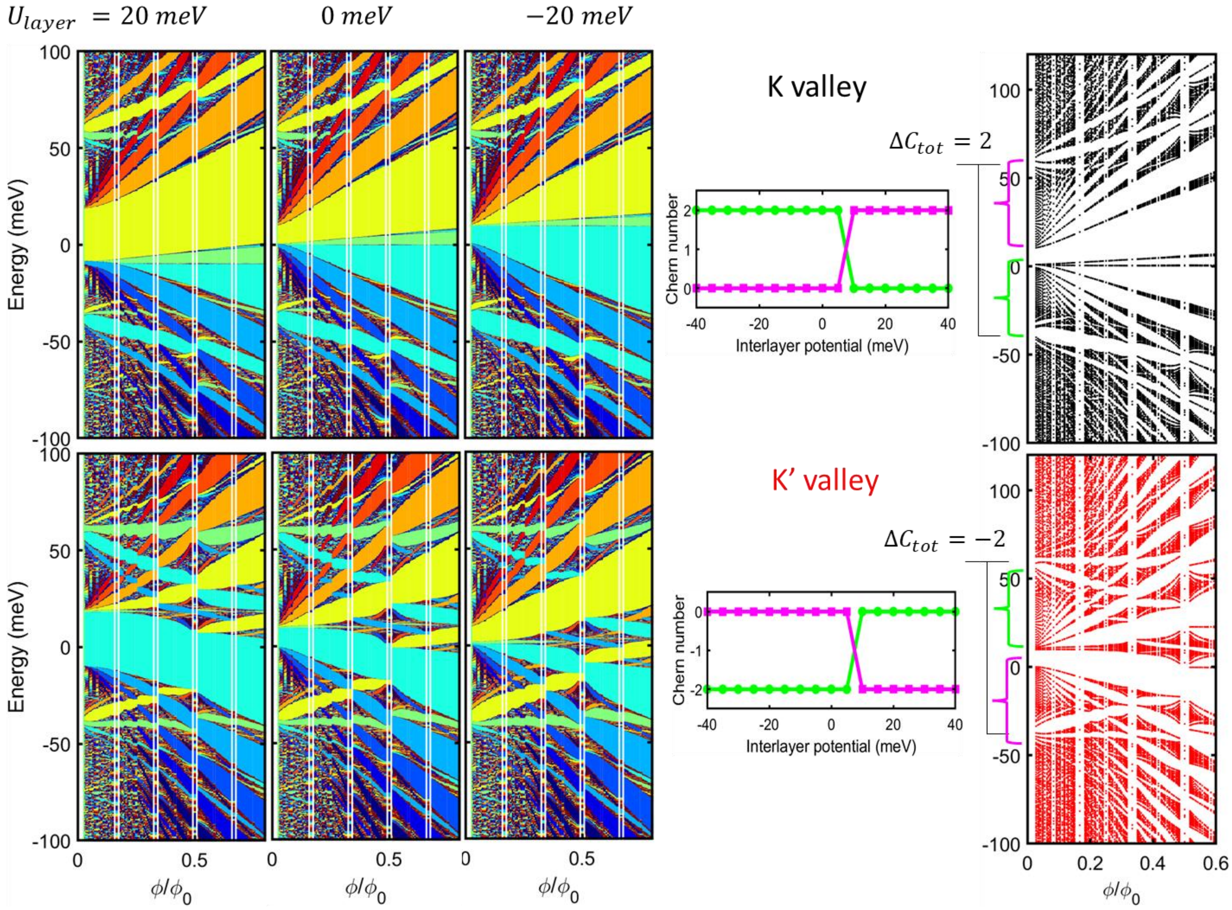

Supplementary Figure 16 | Magneto-spectra for K and K' valleys. At various interlayer potentials, Chern numbers of energy gaps are determined by evaluating $\partial n/\partial\phi$. The Chern numbers of the isolated bands are also evaluated as difference of Chern numbers below and above the bands. In the mid panel, the Chern number of isolated bands at conduction and valence bands are plotted with green and magenta colors, respectively. The total Chern numbers of the two moiré-induced isolated bands ($-4<n/n_0<4$) don't change when the interlayer potential is varied, and the value is clearly valley dependent. In the Hofstadter spectra, we used black (K) and red (K') colors to indicate valleys.

Supplementary Note 6. Discussion on the valley-selective moiré effect and valley-dependent Chern numbers of moiré-induced isolated bands

The valley selective response of the system to the moiré patterns in the presence of an external magnetic field is likely due to the valley selective partial cancellation and reinforcement of the vector potential generated by the magnetic field itself and the virtual strains affecting the system. The vector potential due to a magnetic field breaks time reversal symmetry and leads to asymmetric response of the bands at the K and K' valleys. The virtual strains arise due to second order perturbative hopping effects of the graphene electrons to the hBN atoms and back, leading to time reversal symmetric vector fields for each valley. The pseudomagnetic fields can also result from local strain fields that introduce unequal hopping terms of the electrons at a given lattice site to the neighboring surrounding atoms. These real or virtual strain profiles lead to strong pseudomagnetic fields ranging from a few to a few tens of Teslas at each point in space that have opposing signs for electrons of each valley. However, a real magnetic field has a definitive sign for the electrons in both K and K' valleys.

In the simulation, we find the Chern number of the moiré-induced isolated bands ($-4 < n/n_0 < 4$) have the exact opposite values for each valley (**Supplementary Figure 16**), similar to the observation in ref[4–6]. We attribute this observation is closely related to the observed valley-selective moiré effect. Apparently in the simulation, the Chern numbers for the insulating gap at the conduction band ($n/n_0 = 4$) and for the gap at the valence band ($n/n_0 = -4$) change the sign upon changing the valley quantum number, independent of D field. This in turn leads to the disparate Hofstadter spectra: The effective magnetic moments from the angular momentum of the bands result in the phenomena that the bands of a valley with the anti-parallel band moments and the magnetic field experience more frequent intersections between Hosftadter gaps with opposite Chern values, leading to stronger moiré effects. Moreover, interestingly, based on the simulation, the calculated valley Chern numbers of the induced gaps ($n/n_0 = 4$ and -4) in the valence band seem to suggest that they are quantum valley Hall insulators. The value of the longitudinal resistance for the insulators at $n/n_0 = -4$ at the base temperature appears significantly lower than the expected value from the calculated gap.

This valley selective moiré effect is also observed in Landau levels beyond the lowest ones near the band edges at charge neutrality, namely those that are most directly sensitive to the signs of the applied electric fields. Especially in the limit of small magnetic fields where the Landau levels are closely spaced in energy, it is expected that the Landau level mixing facilitates the propagation of the sublattice/layer/valley-selective moiré effect to higher Landau levels especially when the ground-state solutions are of spin or charge density wave type that favors accumulation of spin-resolved charge in a given sublattice.

Supplementary Note 7. Filling sequence of zeroth Landau level in BBG with interaction

If we draw the energy spectrum (or the filling sequence) of the zero Landau level (ZLL) based on the single particle model (1) in the main text, it is as shown in **Supplementary Figure 17a**, which is different from **Fig. 3e** (**Supplementary Figure 17b**). To explain the difference, let us consider the large D>0 case on the hole side (i.e., the upper left part of **Supplementary Figure 17 a,b**).

The single particle picture of ZLL in BBG with small Zeeman energy expects the filling sequence from $\nu = -4$ should be |Nξσ⟩=|0-↓⟩,|0-↑⟩,|1-↓⟩,|1-↑⟩ for large D>0 case. However, Coulomb interactions change this filling order. Coulomb interaction favors sequential filling of N=0,1 with same spin due to the exchange interaction.[7] i.e. filling different orbitals with same spin,(e.g. |0-↓⟩,|1-↓⟩) has more favorable Coulomb energy than filling same orbital of opposite spin(e.g. |0-↓⟩,|0-↑⟩).

The experimental filling sequence(orbital, valley, spin) of ZLL in our sample was identified by analyzing the Landau fan diagram and n–D sweep. First, we analyzed the orbital nature(N=0 or 1) of the hole side of ZLL at the large D>0 case(i.e. the upper left side of **Fig. 3c**). By observing the ZLL in **Fig. 2a** or **5a**, we can see that filling -4<ν<-3 looks similar to -2<ν<-1(both shows conventional fractional quantum Hall state up to $\phi/\phi_0 = 1/3$), while -3<ν<-2 looks similar to -1<ν<0 (a similar moiré Chern band structure emerged from $\phi/\phi_0 \approx 0.2$). This similarity is due to the same orbital nature of each pair[8] and the single particle calculation in ref (6)[6] showed that the moiré Chern band was developed at a lower magnetic field for N=1 state. This supports the filling order |Nξσ⟩=|0-↓⟩,|1-↓⟩,|0-↑⟩,|0-↑⟩ for large D>0 case in our sample(large D makes valley $\xi = -$ fills before valley $\xi = +$). And in the reference cited in the main text[9], a detailed experimental and theoretical account of the ZLL in the absence of a moiré potential was provided. In ref.(5), the filling sequence of ZLLs according to the size of the interaction is simulated, and it can be seen that in the weak interaction regime, the ZLLs are filled in the order of N=0,0,1,1 as discussed above, and in the intermediate and strong interaction regime, the ZLLs are filled in the order of N=0,1,0,1. By tracking two states feeling moiré potential strongly(|Nξσ⟩=|1-↓⟩,|1-↑⟩) in **Fig. 3c**, we can see that our data is more consistent with the intermediate interaction regime confirmed experimentally in ref.(9).

Additionally, if the magnetic field is further increased, the filling sequence mentioned above can be flipped once more. Since $\Delta_{10}$is proportional to B, while the Coulomb interaction E_C is proportional to $\sqrt{B}$, there will be a point where $\Delta_{10} > E_C$ as the magnetic field increases. In this case, the single particle effect will dominate again, and the electrons will be filled in order |0-↓⟩,|0-↑⟩,|1-↓⟩,|1-↑⟩. However, in our experimental setup, we did not reach the magnetic field where this reversal occurs

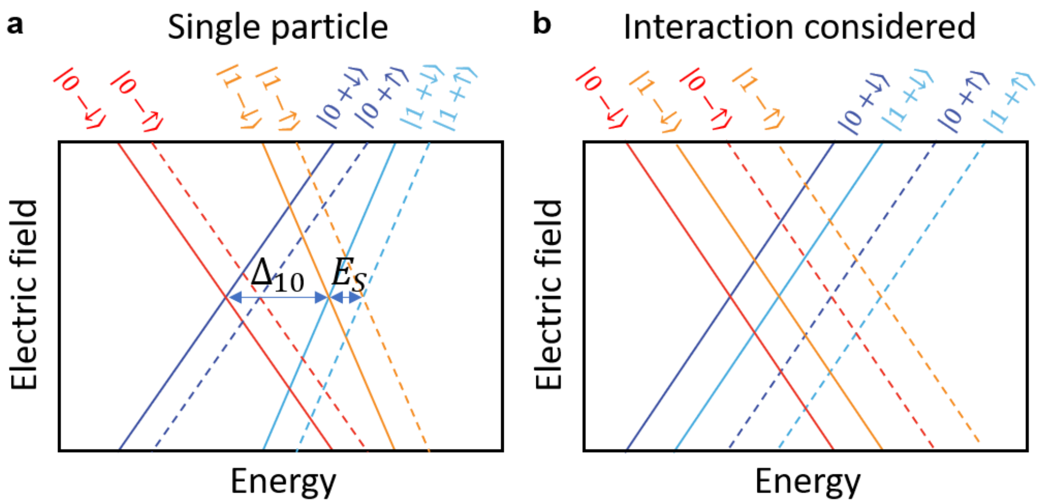

Supplementary Figure 17 | Schematic of ZLL of BBG energy spectrum in purely single particle picture (**a**) and interaction considered (**b**). Note that **b** is purely illustrative since the actual energetics, taking into account interactions, do not simply come out as a straight line. Also, the specific energy gap and slope differences were ignored in **b**.

Supplementary Note 8. Magnetotransport data from other devices.

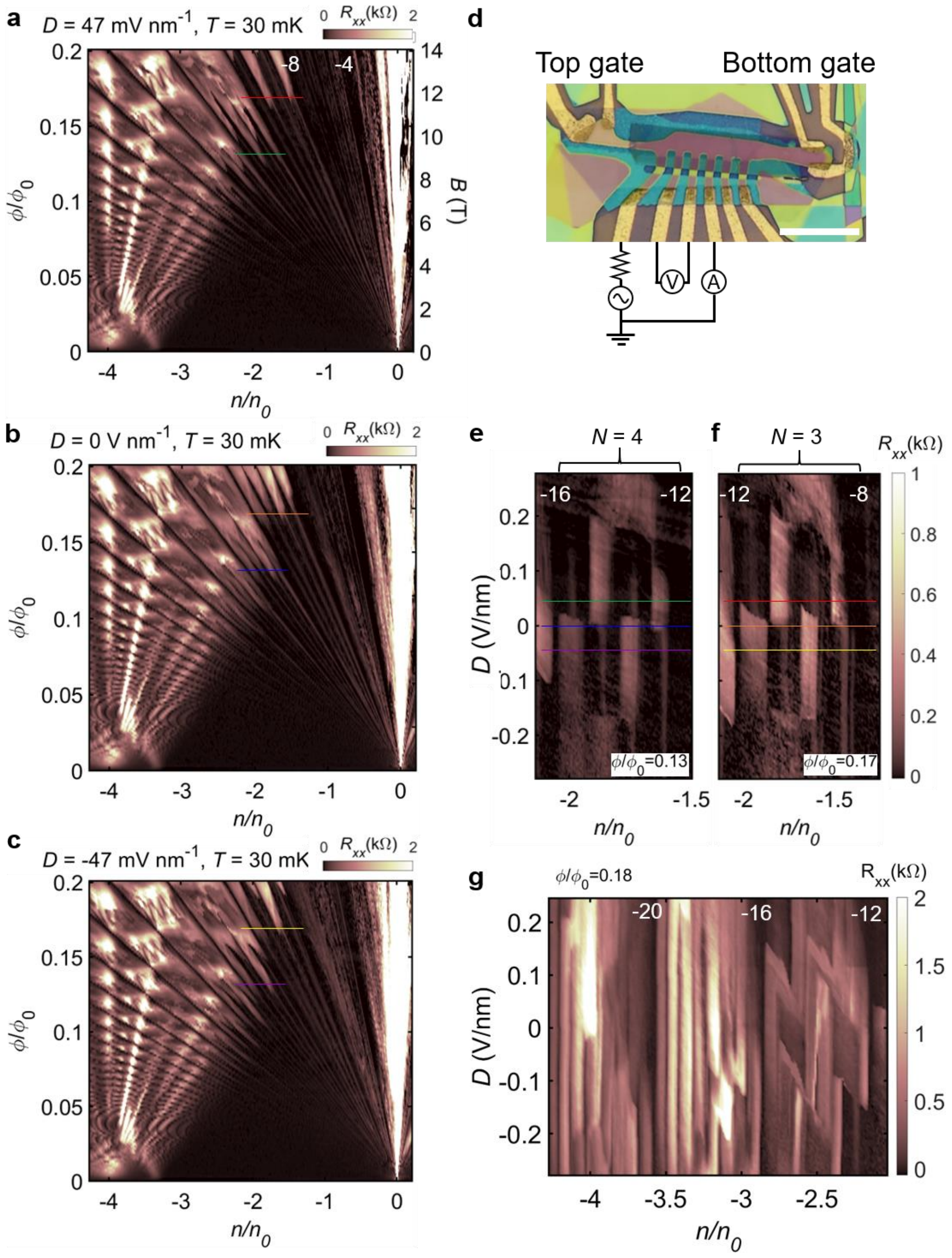

Supplementary Figure 18 | Magnetotransport measurement of 1.37 deg sample. a–c, Landau fan diagram of longitudinal resistance up to B=14T at different vertical displacement fields. All measurements were conducted at T=30mK. Scale bar, 15 µm. **d,** Optical microscope image of the measured device and the schematic of measurement configuration. **e–g,**

Longitudinal resistance R_{xx} as a function of the carrier density n and D field at 8.9T(**e**), 11.75T (**f**), 12.4T (**g**). N denotes the orbital number of the Landau level of BBG and white numbers in **a, d** denote the Landau filling factor. Each colored horizontal line in **e–f** corresponds to the same colored lines in **a–c**.

We fabricated a 1.37 deg aligned device similar to the device used in the main text. (top hBN: 67 nm, bottom hBN: 50 nm) The sample showed similar valley-selective moiré effects in each Landau level and D field tunability of Chern insulators. We observed integer quantum Hall, Chern insulator, and fractional quantum Hall (up to N=3 LL) states in the Hofstadter butterfly pattern of the new sample, but not symmetry broken Chern insulator and fractional Chern insulator states. For the device of the main text, interacting states appeared in the low (less than $1 \times 10^{12}\ cm^{-2}$) carrier density region, but the new device required about three times higher carrier densities to fill the same superlattice filling factor, so we could observe only the high density and low ϕ/ϕ_0 region of Hofstadter patterns in the magnetic field range of our system. We believe that this device will also show interaction driven states if the magnetic field is further increased to access the low carrier density region of the Hofstadter pattern.

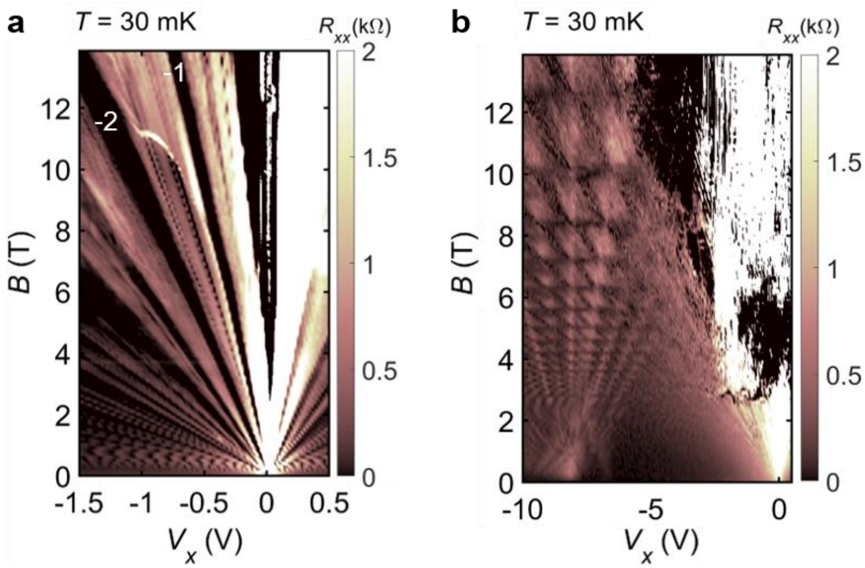

Supplementary Figure 19 | Magnetotransport measurement of other samples. a, Device with gate leakage. Only a small range of carrier density was tuned, and a transition to higher resistance in the ZLL metallic state was observed around B=11T. White number denotes the Landau filling factor of BBG. **b**, Device with bad contact resistance. Fabricated without contact graphite and using a flake with naturally connected trilayer graphene and BBG regions, the contact quality became bad with increasing magnetic field.

Supplementary Note 9. Helical edge states at t=0 insulators.

The left column of **Supplementary Figure 8** below shows the longitudinal resistance (R_L) measurement, and the right column shows the non-local resistance (R_{NL}) measurement. When comparing the two data sets, the state (0,-1) is particularly standing out with unusually high values of R_{NL} over the corresponding R_L.
Our interpretation of the data is based on the following analysis: (We believe this is also consistent with the observation of Sanchez-Yamagishi et al. Nature nanotechnology 12 (2), 118-122 (2017))[10]

1. Compressible state: Intermediate R_L, very low R_{NL}
   - Reasoning: The voltage drops for R_{NL} will be decaying far from the source-drain electrodes.
2. Incompressible state without an edge channel: High R_L, very low R_{NL}
   - Reasoning: The voltage drops for R_{NL} will be decaying far from the source-drain electrodes.
3. Incompressible state with chiral edges: Zero R_L, zero R_{NL}
   - Reasoning: Chiral edges would act as equipotential 1D channels to give nearly zero NL resistance
4. Disordered inhomogeneous phase of multiple states: Intermediate R_L, low R_{NL}
   - Reasoning: Inevitable dissipations occur at every boundary of different states and thus make the situation qualitatively similar to the one of the compressible states.
5. Incompressible state with helical edges: a sizable fraction of the quantum conductance for both R_L and R_{NL}
   - Reasoning: The voltage drops occur near ohmic contacts due to selective mixing of helical 1D channels.

As expected from the above analysis, in **Supplementary Figure 8d**, most compressible states and chiral incompressible states display highly suppressed R_{NL}. Looking at the states of $t = 0$, we can see that they generally have sizable R_{NL}'s. The R_{NL} for (*t*,*s*)=(0,-4) is around 1 kΩ at all magnetic field values. The R_{NL} for (0,-2) is similar in size, except for some regions near $\phi/\phi_0 \sim 0.2, 0.4$. For (0,-1), while the longitudinal resistance peak is the smallest of the three, R_{NL} shows the largest value, approaching $h/4e^2$ at high magnetic fields, which is the expected value to be measured for states with a single helical edge when the NL configuration is used as shown in **Supplementary Figure 8b**, based on the Landauer–Büttiker formalism. This strongly suggests the existence of non-local helical transport at (0,-1). In the same vein, the state at (0,-2) near $\phi/\phi_0 \sim 0.4$ also hints on a helical state.

For the question about the nature of the state that can harbor a helical edge in this system, we think it is a state having the valley-dependent Chern numbers with opposite signs at K and K', with suppressed intervalley scattering. Actually, our calculation of the valley-specific Hofstadter spectra in **Supplementary Figure 16** shows that, in ZLL, where the observed (0,-1) state resides, the K valley has a Chern number of -2 over a wide range of energy (the absence of states for K) while the K' valley displays moiré-induced subbands with many different Chern numbers. From the fact that the total Chern number of the observed (0,-1) state should be 0 means that an energy gap opens at the Fermi level with the Chern number of +2 in the K' valley. We originally named

this state as a QVH state, in which the valleys have edge channels with opposite chirality, forming helical edge states.

We note that, since the spectra in **Supplementary Figure 16** are limited to the spin degenerate case, a spin hall state or a complex spin–valley magnetic state are not considered and thus cannot be ruled out given the spin degeneracy is to be broken in the real system. Nonetheless, we think that the valley-dependent Chern number provides a crucial mechanism in the formation of the helical edge state.

Supplementary Note 10. Temperature dependence of n/n_0=-4 satellite peak at zero magnetic field.

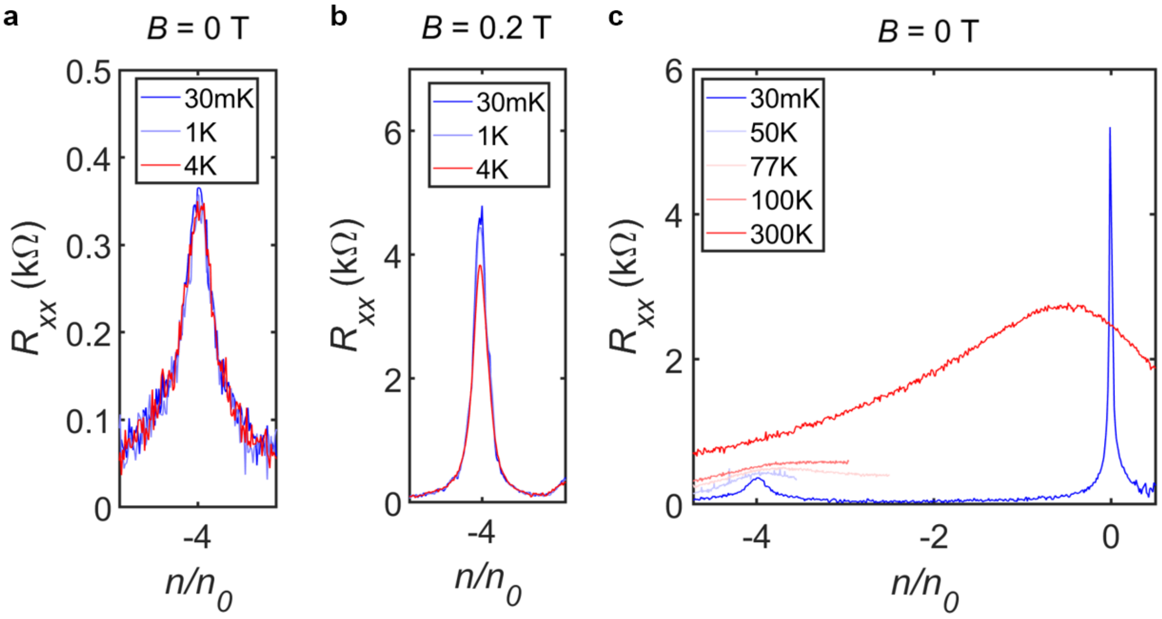

Supplementary Figure 20 | Temperature dependent longitudinal resistance measurement at *D*=0. a, R_{xx} peak of $n/n_0 = -4$ at *B*=0T up to *T*=4K. **b**, R_{xx} peak of $n/n_0 = -4$ at *B*=0.2T up to T=4K. In the presence of a magnetic field, the $n/n_0 = -4$ peak shows insulating behavior. **c**, High temperature R_{xx} measurements at B=0 up to T=300K

Theoretical papers on BBG–hBN aligned devices predict either a full gap or a DOS minimum at the secondary Dirac point resulting from the moiré pattern, depending on the parameters used. A recent paper on a small angle twisted BBG–hBN system shows that the band structure with a DOS minimum is in better agreement with experimental results. To verify whether the satellite peak in our device is a gap or a DOS minimum, we performed transport measurements at higher temperatures (**Supplementary Figure 20c**). Up to 4 K, where most of the experiments in the main text were performed, it is difficult to distinguish whether the satellite peak is metallic or insulating in the absence of a magnetic field. (**Supplementary Figure 20a**) However, as we increase the temperature beyond a few tens of K, we find that the resistance of the satellite peak increases with it, confirming that it is a DOS minimum rather than a full gap at n/n_0=-4 in our sample.

Supplementary Note 11. Characterization of graphite contacts.

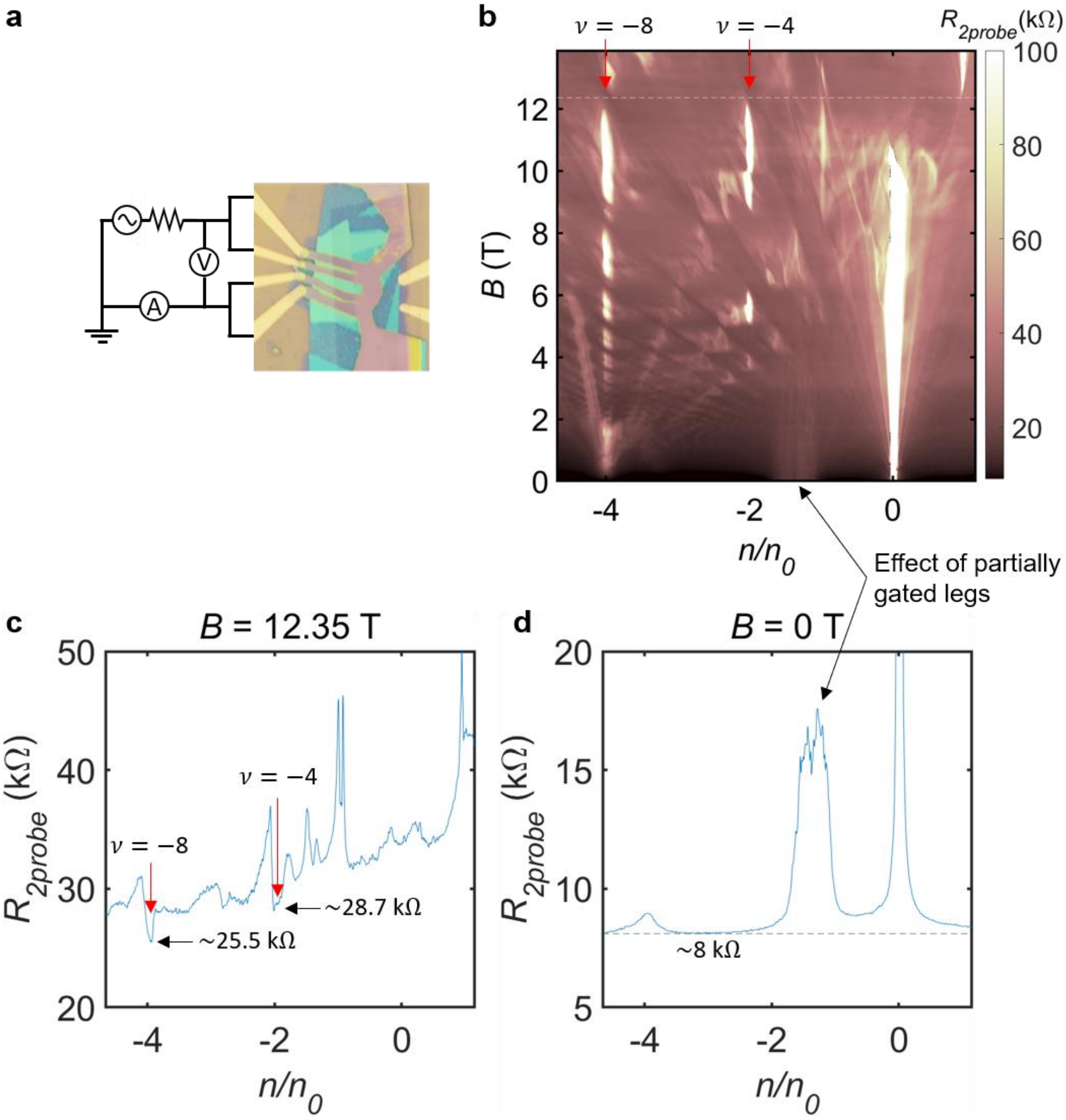

Supplementary Figure 21 | Contact resistance characterization. a, Measurement configuration of two-probe resistance. Two legs are paired in parallel and the series resistance of the two pairs was measured. This makes the measured quantity approximate the resistance coming from a single leg. **b,** Landau fan diagram of 2-probe resistance at T = 30 mK, D=116 $\mathrm{mV/nm^{-1}}$. The colormap is truncated at both ends. **c,** Line cut at B=12.35T ($\phi/\phi_0 = 0.5$, white dashed line in **b**)。 The red arrows in **b**,**c** indicate Landau filling factor -4 and -8 points. **d**, Line cut at B=0T. The 8 kΩ line is shown as a black dashed line. The black arrows denote the area affected by the partially gated legs.

To characterize the contact resistance, we conducted an additional two-probe magnetoresistance measurement at T = 30 mK. At zero field, the lowest value of the two-probe resistance was measured to be about 8 kΩ, which directly gives an approximate contact resistance due to the small four-probe resistance. At high magnetic fields, we compared the resistance values of the ν=-4 and ν=-8 IQHEs, which are among the strongest features in the single particle picture, and found them to be about 28.7 kΩ and 25.5 kΩ, respectively. We analyzed that the difference, about 3.2 kΩ, came from the difference in conductance of the edge states ($(h/e^2)/8 \sim 3.2$ kΩ), so we estimated the residual resistance to be about 22 kΩ. Subtracting 2 kΩ of cryostat wire resistance from this, the contact resistance is approximately 6 kΩ at zero field and 20 kΩ at 12.35 T. (This is not much different at 14 T) Although it increases by about a factor of 3, we found the graphite contact remained good enough that even the two-probe measurement was able to reproduce most of the detailed features observed in the four-probe measurement.

Supplementary References

1. Maher, P. *et al.* Evidence for a spin phase transition at charge neutrality in bilayer graphene. *Nature Phys.* **9**, 154–158 (2013).
2. Pan, C. *et al.* Layer Polarizability and Easy-Axis Quantum Hall Ferromagnetism in Bilayer Graphene. *Nano Lett.* **17**, 3416–3420 (2017).
3. Li, H. *et al.* Electrode-Free Anodic Oxidation Nanolithography of Low-Dimensional Materials. *Nano Lett.* **18**, 8011–8015 (2018).
4. Zhang, Y.-H., Mao, D., Cao, Y., Jarillo-Herrero, P. & Senthil, T. Nearly flat Chern bands in moiré superlattices. *Phys. Rev. B* **99**, 075127 (2019).
5. Chittari, B. L., Chen, G., Zhang, Y., Wang, F. & Jung, J. Gate-Tunable Topological Flat Bands in Trilayer Graphene Boron-Nitride Moiré Superlattices. *Phys. Rev. Lett.* **122**, 016401 (2019).
6. Ghorashi, S. A. A. *et al.* Topological and Stacked Flat Bands in Bilayer Graphene with a Superlattice Potential. *Phys. Rev. Lett.* **130**, 196201 (2023).
7. Barlas, Y., Côté, R., Nomura, K. & MacDonald, A. H. Intra-Landau-Level Cyclotron Resonance in Bilayer Graphene. *Phys. Rev. Lett.* **101**, 097601 (2008).
8. Spanton, E. M. *et al.* Observation of fractional Chern insulators in a van der Waals heterostructure. *Science* **360**, 62–66 (2018).
9. Hunt, B. M. *et al.* Direct measurement of discrete valley and orbital quantum numbers in bilayer graphene. *Nat Commun.* **8**, 948 (2017).
10. Sanchez-Yamagishi, J. D. *et al.* Helical edge states and fractional quantum Hall effect in a graphene electron–hole bilayer. *Nature Nanotech.* **12**, 118–122 (2017).
